# The Versatility of Lateral Chest Wall Perforator Flaps in Immediate and Delayed Breast Reconstruction: Retrospective Study of Clinical Experience with 26 Patients

**DOI:** 10.1177/22925503211051110

**Published:** 2021-12-20

**Authors:** Helene Retrouvey, Mary-Helen Mahoney, Brian Pinchuk, Waqqas Jalil, Ron Somogyi

**Affiliations:** 1 7938 University of Toronto, 8613 North York General Hospital, Toronto, Ontario, Canada

**Keywords:** breast reconstruction, flap, perforator, oncoplastic, breast conserving surgery, mastectomy

## Abstract

**Rationale:** Lateral chest flaps represent versatile reconstructive options, especially valuable in times of global healthcare resource restriction. In this series, we present our experience with the use of lateral chest wall flaps in both immediate and delayed reconstruction from both breast conserving and mastectomy surgery. **Methods:** A retrospective cohort study of patients who had undergone a lateral chest wall flap for immediate or delayed breast reconstruction of a lumpectomy or mastectomy defect was performed. Data collected consisted of patient demographics, procedure type, tumor/oncological characteristics, as well as postoperative complications. **Findings:** Between September 2015 and April 2021, 26 patients underwent breast reconstruction using a lateral chest wall flap. Fifteen patients (58%) underwent immediate reconstruction (9 lumpectomy; 6 mastectomy) and 11 (42%) underwent delayed breast reconstruction. All flaps survived, though 1 patient required partial flap debridement following venous compromise hours after surgery. There were no incidences of hematoma, seroma, infection, or wound healing delay at either the donor site or breast. There was one positive margin which occurred in a mastectomy patient. **Significance:** This study describes the use of lateral chest wall flaps in a wide variety of reconstructive breast surgery scenarios. This technique can be safely performed in an outpatient setting and does not require microvascular techniques. Review of our outcomes and complications demonstrate that this is a safe and effective option. Our experience is that this is an easy to learn, versatile flap that could be a valuable addition to the surgeon's arsenal in breast reconstruction.

## Résumé

**Justification:** Les lambeaux thoraciques latéraux constituent une option de reconstruction polyvalente, particulièrement utile par des temps de restriction globale des ressources en soins de santé. Dans cette série, nous présentons notre expérience de l’utilisation du volet latéral de paroi thoracique aussi bien en cas de reconstruction immédiate que différée pour les chirurgies de conservation du sein et pour les mastectomies. **Méthodes:** Une étude de cohorte rétrospective de patientes ayant subi un lambeau latéral de la paroi thoracique pour reconstruction immédiate ou différée du sein pour lumpectomie ou mastectomie a été réalisée. Les données collectées ont inclus les caractéristiques démographiques des patientes, le type de procédure, les caractéristiques de la tumeur/oncologiques ainsi que les complications postopératoires. **Résultats:** Entre septembre 2015 et avril 2021, vingt-six patientes ont subi une reconstruction du sein avec lambeau latéral de la paroi thoracique. Quinze patientes (58%) ont eu une reconstruction immédiate (9 lumpectomies, 6 mastectomies) et onze (42%) ont eu une reconstruction différée du sein. Tous les lambeaux ont survécu bien qu’une patiente ait nécessité un débridement partiel du lambeau après un trouble veineux, quelques heures après l’intervention chirurgicale. Il n’y a pas eu de survenue d’hématome, de sérome, d’infection ou de retard de cicatrisation au niveau du site donneur ou du sein. Des marges positives sont survenues chez une patiente mastectomisée. **Signification:** Cette étude décrit l’utilisation de lambeaux latéraux de la paroi thoracique dans une grande variété de scénarios de chirurgie mammaire reconstructrice. Cette technique peut être employée de manière sécuritaire dans un cadre ambulatoire et ne nécessite pas de recours à des techniques microvasculaires. L’analyse de nos résultats et des complications démontre qu’il s’agit d’une option sécuritaire et efficace. Selon notre expérience, cette technique est facile à apprendre; le lambeau polyvalent pourrait être un supplément intéressant dans l’arsenal du chirurgien pour la reconstruction mammaire. **Mots-clés:** reconstruction mammaire, lambeau, perforateur, oncoplastique, chirurgie de conservation mammaire, mastectomie

## Background

Breast conservation surgery (BCS) is the most common treatment of breast cancer allowing for improved quality of life, aesthetic and functional benefits as compared to mastectomy.^1–4^ In patients with small breasts, large tumors or for tumors located in aesthetically sensitive areas of the breast, significant deformities can occur following BCS.^5^ The field of oncoplastic surgery has extended the indications for breast conservation for many patients by providing techniques to address anticipated deformities.^6,7^ These oncoplastic techniques are generally divided into tissue rearrangement and tissue replacement.^8^ Commonly performed tissue rearrangement techniques include volume displacement with dermo-glandular breast-based pedicles and breast reduction pattern skin excisions; these are excellent in patients with adequate breast volume and/or ptosis.

Tissue replacement technique involves the use of local flaps. The most common option is the pedicled latissimus dorsi musculocutaneous flap, but this flap is associated with considerable morbidity.^9,10^ One of the most versatile, recently described, group of autologous local flaps are the lateral chest wall perforator flaps.^11–14^ Lateral chest wall flaps have a number of distinct advantages including preservation of the underlying latissimus muscle, a relatively well concealed scar, avoiding a contralateral balancing procedure and the ability to perform the resection and reconstruction in the same position.^15^ Possible complications of lateral chest wall flaps include seroma or hematoma, delayed wound healing, wound infection, fat necrosis, partial or complete flap loss, need for reexcision or competition mastectomy secondary to a positive margin.^8,16^ This group of flaps include the lateral thoracic artery perforator flap, the lateral thoracodorsal flap, the lateral intercostal artery perforator (LICAP) flap, or the thoracodorsal perforator flap.^17–22^ These flaps are available in most patients and can be used to fill most defects of the superior, lateral, and inferior breast. Importantly, they are described with minimal donor site morbidity and the ability to maintain the blood supply to the latissimus dorsi for future reconstruction if needed.

With time, we have further realized the increased versatility of lateral chest wall flaps in delayed lumpectomy reconstruction, salvage from failed mastectomy reconstruction and more complex immediate lumpectomy reconstruction. Finally, where lumpectomy is not possible and limited options exist for standard immediate mastectomy reconstruction, the breast mound can be reconstructed with the use of a large lateral chest wall flap in conjunction with deepithelialized breast skin.^18^ This experience with lateral chest wall flaps has proven specifically valuable in our health care systems with resource limitations and can be particularly valuable in times of global healthcare resource restriction.

In this series, we aim to present our 6-year experience with the use of lateral chest wall flaps in both immediate and delayed reconstruction in order to evaluate the safety of these flaps in both breast conserving and mastectomy surgery. We also describe our surgical technique, encouraging surgeons to include lateral chest flaps to their armamentarium, especially in a resource limited setting.

## Methods

After obtaining institutional review board approval, a retrospective cohort of patients who had undergone a lateral chest wall flap at a single institution for immediate or delayed reconstruction of a lumpectomy or mastectomy defect between 2015 and 2021 was identified. Eligible patients were identified through case log reviews by plastic surgeons.

Data was collected through chart review by each of the 3 surgeons. This included patient demographics as well as surgical and oncological data. Patient age, preoperative body mass index, and preexisting comorbidities were collected. Tumor characteristics, type of reconstruction (immediate vs delayed), and postoperative complications were also collected. Patients were evaluated immediately postoperatively, at 1, 4, and 12 weeks and at 6 and 12 months postoperatively. Complications at any time point were recorded through chart review and included in our analysis. Patients were also followed long term per Cancer Care Ontario recommendations, and any recurrences were noted.

### Statistical Analysis

Summary statistics were generated for all collected data points. Descriptive statistics were produced (frequencies and percentages).

### Surgical Technique

For immediate reconstruction (see Example cases 1–4 [Figures 2 to 5]), all patients were assessed preoperatively by the multidisciplinary breast care team. In-depth discussion was carried out between the breast oncological surgeon and plastic surgeon to plan the oncological resection and discuss the anticipated defect. At our institution, alloplastic, pedicled, and free flap options are considered for all patients. On the day of surgery, the planned resection is marked with both the Oncologic and Plastic surgeon present.

**Figure 1.**
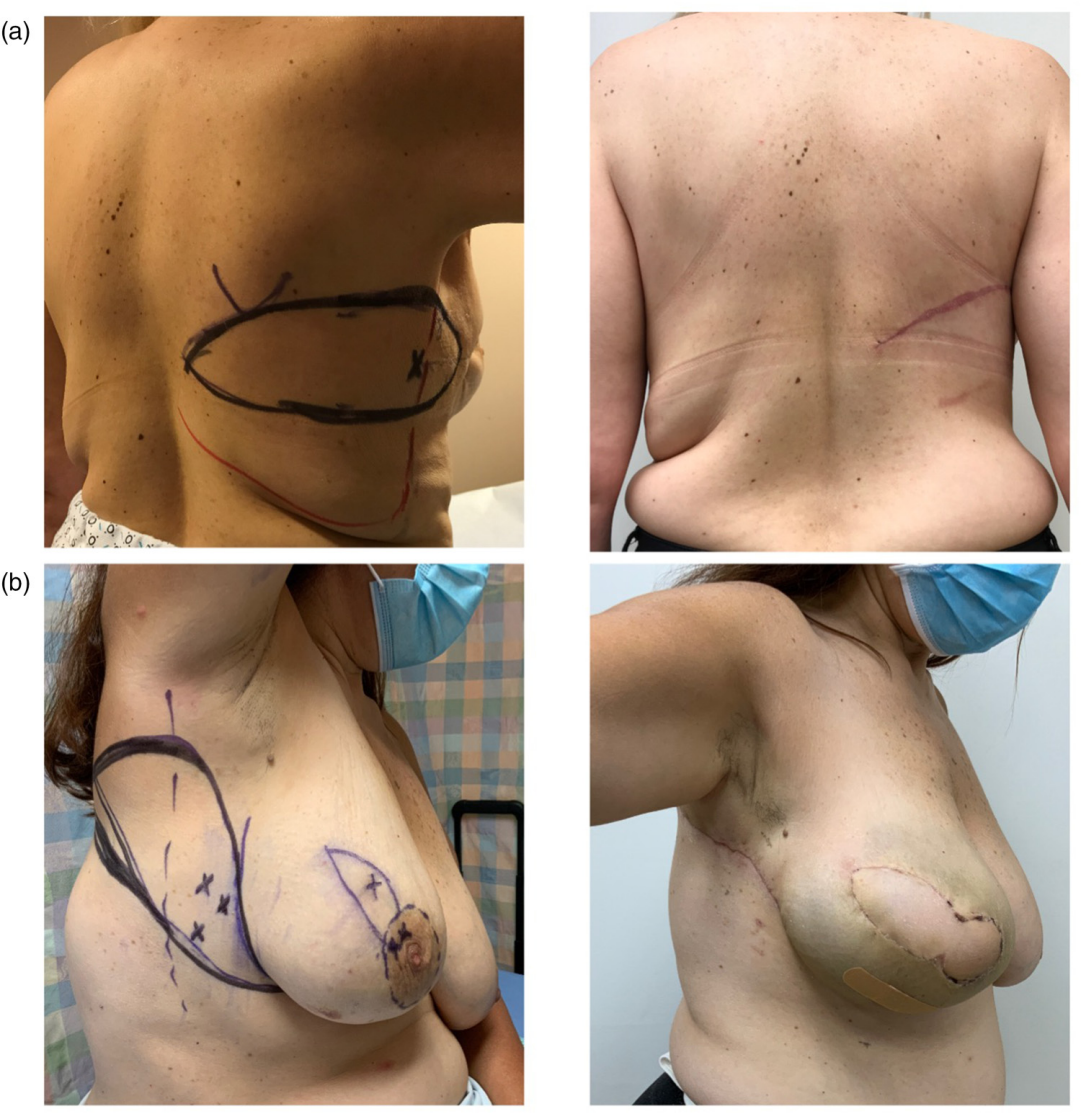
(a) Traditional markings for the horizontal orientation of the lateral chest wall flap; (b) the lateral chest wall flap modified orientation incorporates lateral chest/axillary skin excess, limiting the posterior scar.

**Figure 2.**
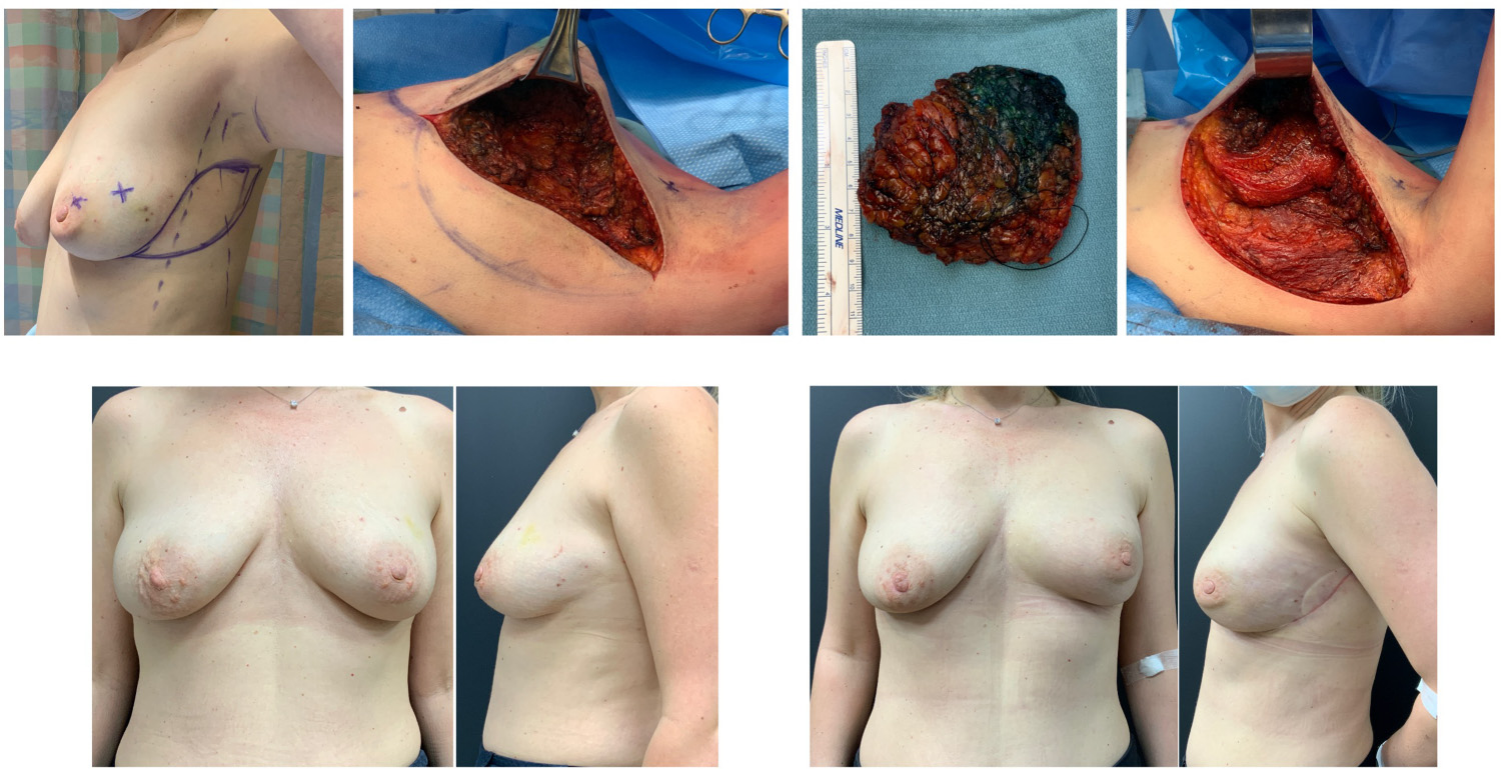
Example case 1: Left lumpectomy requiring immediate tissue replacement.

Fifty-year-old female with left upper outer quadrant invasive duct cancer and adjacent ductal carcinoma in situ. A modified lateral chest wall flap is drawn preoperatively. The lumpectomy is performed through the lateral breast incision (represents the anterior edge of the flap) to remove a specimen that weighs 117 g. The large lateral breast defect is shown. The flap is elevated and transposed to fill the defect. Patient is shown preoperatively and 4 months after lumpectomy with immediate lateral chest wall flap reconstruction (2 months after adjuvant radiation).

**Figure 3.**
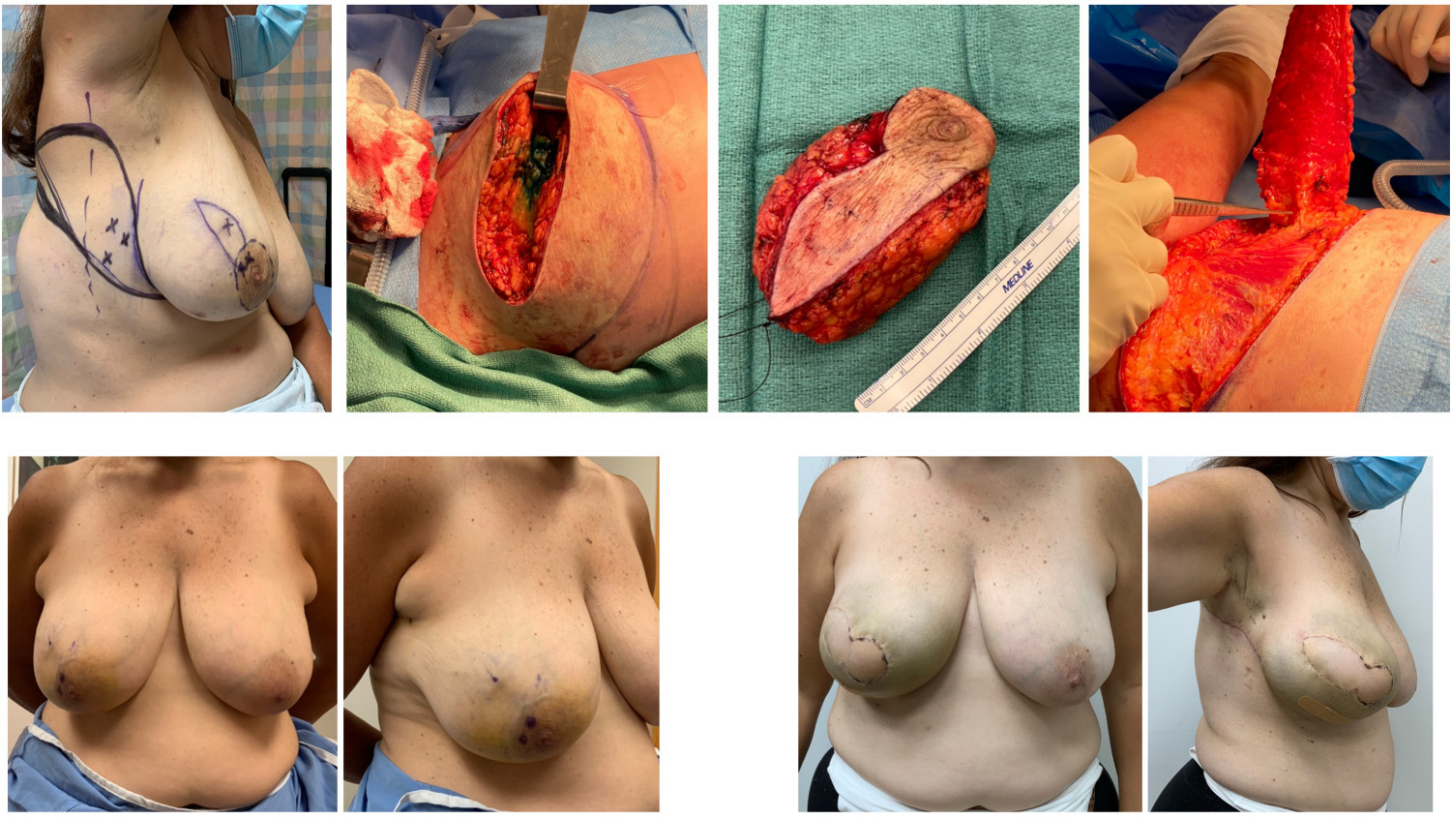
Example case 2: Right lumpectomy requiring immediate tissue & skin replacement.

Forty-six-year-old female with central breast invasive duct cancer and adjacent ductal carcinoma in situ. The area of involvement is tethering the nipple/areolar complex and upper outer quadrant skin. A modified lateral chest wall flap is drawn preoperatively with 3 perforators heard on portable Doppler. The lumpectomy is performed to remove a specimen that includes the affected skin and weighs 152 g. The flap is elevated with a single visible perforator identified is transposed to fill the defect. Partial flap skin is used to replace breast skin and nipple areolar complex. Patient is shown before surgery during marking and 4 weeks after lumpectomy and immediate lateral chest wall flap reconstruction.

**Figure 4.**
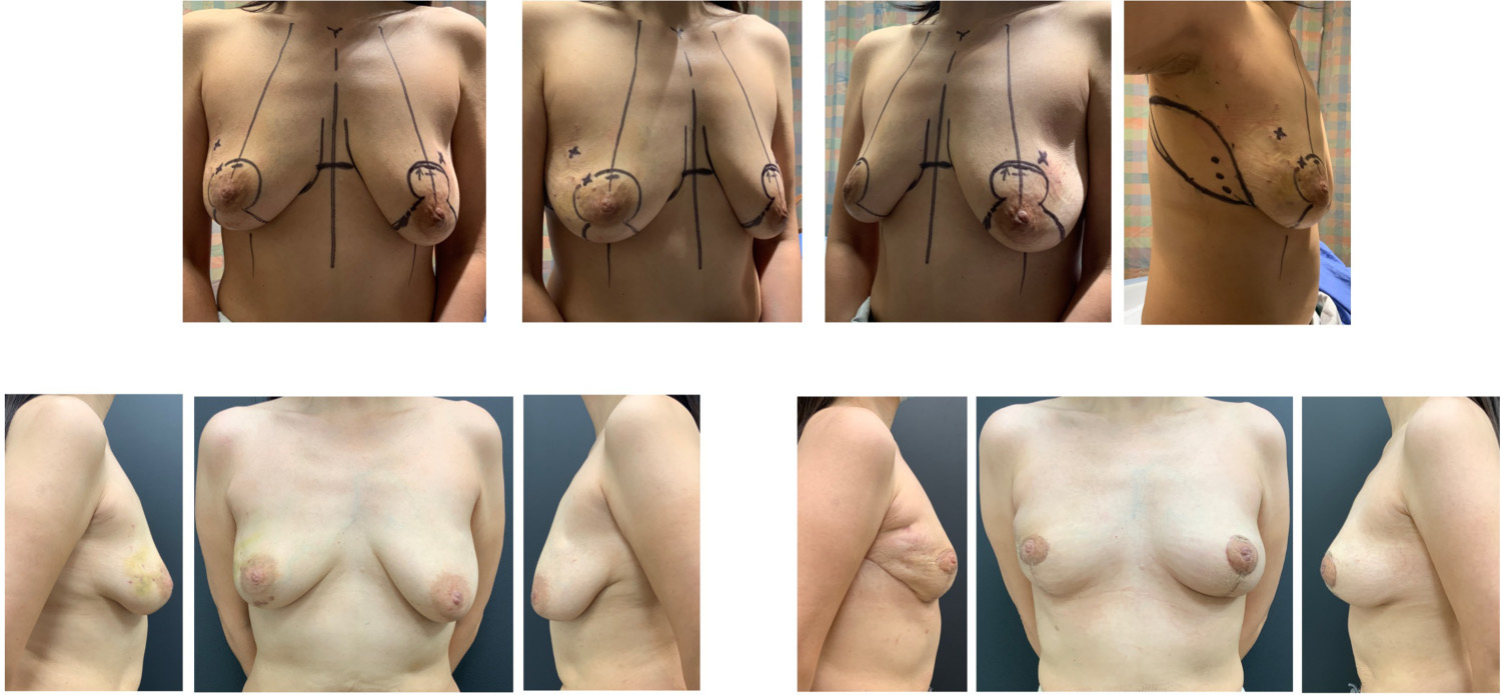
Example case 3: Bilateral lumpectomy requiring bilateral circum-vertical tissue rearrangement and right breast tissue replacement.

Fifty-year-old female with bilateral breast cancer. The smaller area of invasive duct cancer of the left breast was managed with standard oncoplastic lumpectomy and immediate circum-vertical tissue rearrangement. The larger excision of right breast invasive duct cancer and adjacent ductal carcinoma in situ required similar circum-vertical tissue rearrangement but also required tissue replacement with lateral chest wall flap to match the volume of contralateral left side. The patient is shown marked for bilateral circum-vertical mastopexy tissue rearrangement and right modified lateral chest wall flap. Patient is then shown preoperatively and 5 months postoperatively.

For delayed reconstruction (see Example cases 5 and 6 [Figures 6 and 7]), the area of deformity was assessed by the plastic surgeon, considering both volume and skin requirements. The lateral chest wall flap was then planned.

**Figure 5.**
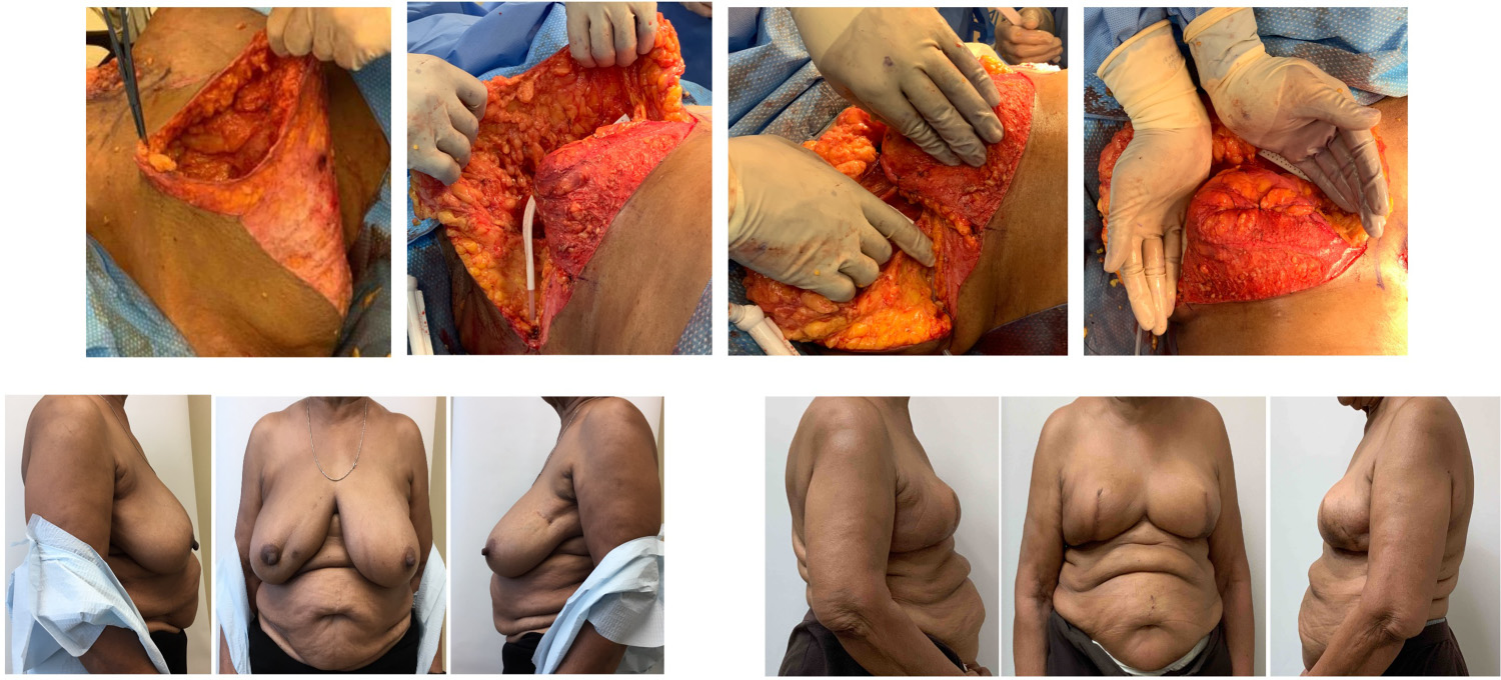
Example case 4: Immediate right breast mastectomy reconstruction.

Seventy-year-old female with a large area of right breast DCIS that was recommended for mastectomy. She underwent a right wise pattern mastectomy with preservation of deepithelialized skin and right lateral chest wall flap for volume restoration. She underwent a concurrent left balancing breast reduction using the same skin pattern. Patient is shown preoperatively and 2 months postoperatively.

**Figure 6.**
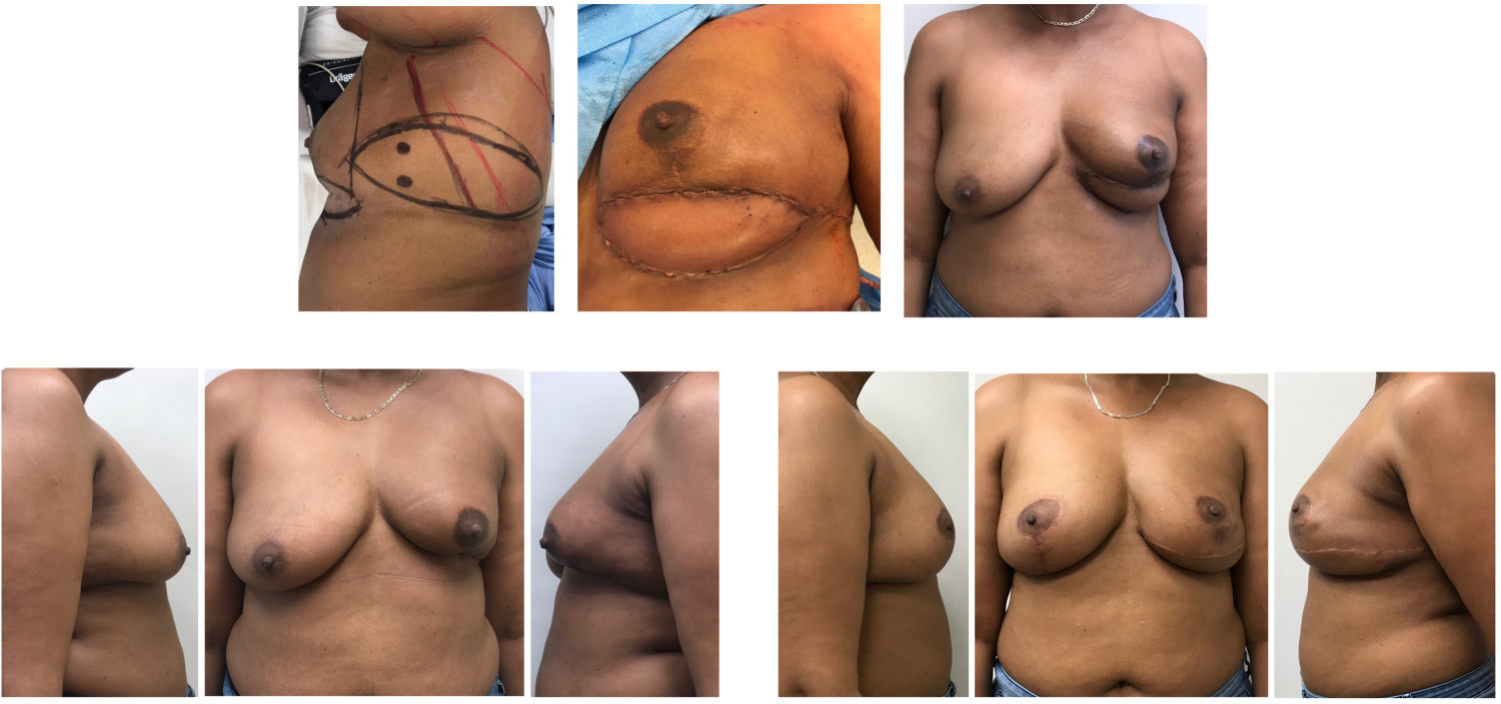
Example case 5: Delayed left breast reconstruction.

Forty-five-year-old female with left breast deformity. She underwent breast conserving therapy for a lower quadrant breast cancer 2 years prior to presentation to us for reconstruction. She presents with deficiency in lower pole volume and a tight, elevated inframammary fold. The area of deficiency is marked along the inframammary fold and a traditional lateral chest wall flap is marked along the adjacent lateral chest wall. The flap is elevated and rotated 180° to resurface the inframammary fold and lower breast tissue. Patient is shown during preoperative markings, intra-operatively and 3 weeks after flap reconstruction (top panel). Patient is also shown preoperatively and after second stage flap recontouring and contralateral balancing breast reduction (bottom panel).

**Figure 7.**
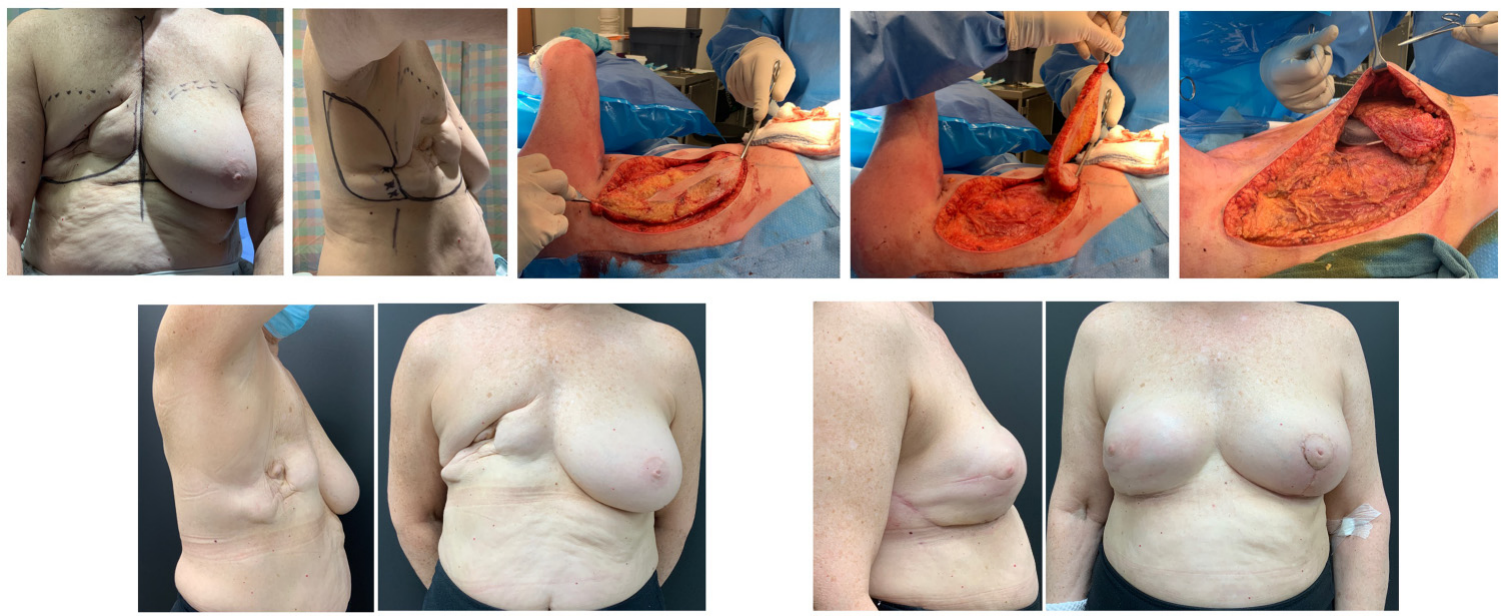
Example case 6: Delayed right breast mastectomy reconstruction.

On the day of surgery, the flap is marked with the patient in the preoperative area. With the patient in the lazy lateral decubitus position, a handheld Doppler is used to locate several chest wall perforators along the lateral breast margin at the level of the fourth to sixth rib. The flap is then marked to incorporate the maximum number of perforators while considering skin elasticity by pinch test. In our earliest cases, the flaps were designed horizontally to place the scar within the lateral and posterior bra-line (Figure 7(a)).^15^ In recent cases, the markings were made as described in the modification of the LICAP flap by Meybodi et al (Figure 7(b)).^23^

Seventy-one-year-old female with right breast invasive duct cancer that was treated at another institution with nipple sparing mastectomy and immediate implant reconstruction. Patient is shown 2 years after implant removal for infection. Thin, tethered skin and nipple/areolar complex requires improved soft tissue coverage to allow 2-stage tissue expander and implant reconstruction. A modified lateral chest wall flap is drawn preoperatively with 3 perforators heard on portable Doppler. The mastectomy flaps are elevated through the lateral breast incision and the pocket for the tissue expander is defined. The flap is then elevated, deepithelialized, and transposed to line the thin area of the mastectomy flaps. A tissue expander is then placed beneath the flap within the breast pocket. The patient is shown preoperatively and 3 months after second stage replacement of tissue expander for implant and contralateral balancing mastopexy.

In the operating room, patients were positioned on a beanbag with pressure points carefully padded. Upon induction of general anesthesia, patients were transferred into a lazy lateral decubitus position with the arm on the flap side prepped and draped and placed on an elevated mayo stand allowing visualization of the affected breast and lateral chest.

In cases where skin excision was not required for oncological purposes, the breast surgeon accessed the tumor via the lateral breast incision, which also represents the anterior edge of the flap. When breast skin or nipple areola complex excision was indicated, tumor excision was performed through a more direct approach, excising the necessary skin.

Following tumor excision, the tumor bed is clipped with 4 titanium clips. The flap is then elevated from lateral to medial at the level of the muscle fascia. Dissection is carried out with electrocautery until 1 cm from the Dopplered perforators. At this point, the remaining tissue around the perforators is carefully dissected to allow the flap to rotate into the defect without tension. Skeletonization of the perforator is generally not required. As such, the perforator is only occasionally visualized. The flap was shaped as needed into the defect and secured using absorbable sutures. The flap was deepithelized as necessary. The donor site was closed in multiple layers over a suction drain.

## Results

Between September 2015 and April 2021, 26 patients underwent breast reconstruction using a lateral chest wall flap. Patient demographics are outlined in Table 1. Twenty-six flaps were used in 26 patients. Of these, 15 (58%) were immediate and 11 (42%) were delayed. Of the immediate reconstruction patients, 9 were treated with lumpectomy and 6 were treated with mastectomy. Of the 6 patients that were treated with mastectomy, all underwent contralateral balancing at the time of flap reconstruction.

**Table 1.** All Patient Demographics, Tumor Characteristics, and Complications.

Demographics			**%**
	Patients (n)	26	
	Age (years)	57	
	Body mass index (kg/m^2^)	28	
Comorbidities (n)			
	Hypertension	1	3.8
	Diabetes		0.0
	Smoking	1	3.8
Tumor characteristics (n)			
	Tumor side right	14	53.8
	Tumor side left	10	38.5
	Tumor side bilateral	2	7.7
	Immediate breast reconstruction	15	57.7
	Delayed breast reconstruction	11	42.3
	Skin involvement	11	42.3
Complications (n)	Hematoma	0	0.0
	Seroma	0	0.0
	Infection	0	0.0
	Donor site complication	0	0.0
	Fat necrosis	2	7.7
	Flap loss	0	0.0
			
	Revision/Second stage	4	15.4
			
Follow-up (months)	All patients (n = 26)	8.5	
	Immediate reconstruction (n = 15)	7.9	

All flaps survived except for one flap that demonstrated significant venous compromise several hours after surgery. In the operating room, venous compromise could not be improved and the distal 2/3 of the flap was debrided. No obvious patient comorbidity or surgical mishap was identified in this patient. There were no incidences of hematoma, seroma, infection, or wound healing delay at either the donor site or breast. Four (15.4%) patients underwent revision surgery to improve the shape of the reconstructed breast or for better balancing. Average follow-up of the entire cohort was 8.5 months while average follow-up of the immediate reconstruction group was 7.9 months.

Four patients underwent revision surgery for improved cosmesis and balancing. Revisions included fat grafting and balancing mastopexy/reduction and were completed on average 6.75 months after flap reconstruction (range 4-9 months). Formal balancing reduction performed at the time of reconstruction only occurred in the mastectomy patients for which insufficient volume is available using a chest wall flap alone.

Of the immediate reconstruction patients, 9 (60%) of patients had both invasive disease and ductal carcinoma in situ (DCIS), 3 patients (13%) had invasive disease only, and 2 (20%) had DCIS only. One patient had a malignant phyllodes tumor. Eight patients (53%) had right sided tumors, 5 patients (33%) had left sided tumors, and 2 patients had bilateral tumors. Average tumor size was 33.8 mm and average resection specimen weight in the lumpectomy patients was 113 g. Most tumors were at least partially located in the upper lateral and/or central aspects of the breast (Table 2).

**Table 2.** Immediate Reconstruction Patient Surgery Details and Tumor Characteristics.

Immediate reconstruction	Patients (IBR)	15	
	Neo-adjuvant chemotherapy	4	27%
	Tumor size (mm)	33.8	
	Mastectomy	6	40%
	Lumpectomy	9	60%
	Lumpectomy resection specimen weight (g)	113.35	
Tumor side	Right	8	53%
	Left	5	33%
	Bilateral	2	13%
Tumor location	Upper lateral	11	73%
	Upper medial	4	27%
	Lower lateral	3	20%
	Lower medial	3	20%
	Central	7	47%
	Crossover	8	53%
Tumor type	Invasive Dx	2	13%
	DCIS	3	20%
	Invasive Dx + DCIS	9	60%
	Other	1	7%
	Positive margin	1	7%

Additional oncologic variables are outlined in Table 2.

Only one positive margin occurred (7%). This occurred in one of our mastectomy patients and was managed with radiotherapy.

## Discussion

Tissue replacement techniques extend the possibilities for breast conservation.^18^ Patients with large tumors, inadequate breast tissue for rearrangement, those that want to maintain their breast volume, those that do not want a balancing procedure on the contralateral side, and those that require significant skin and/or nipple excision could benefit from reconstruction using tissue replacement.^5^ Additionally, in salvage scenarios or mastectomy patients that are not good candidates for alloplastic or free flap reconstruction, local tissue replacement can provide additional reconstructive options. Lateral chest wall flaps are therefore ideal based on oncological parameters and patient preference, though there are no ideal candidate in terms of patient characteristics. This study describes the use of lateral chest wall flaps in a wide variety of reconstructive breast surgery scenarios. For the majority of our sample, it was a safe and effective option given the low complication rate within our follow-up period. Our experience is that this is an easy to learn, versatile flap that could be a valuable addition to the surgeon's arsenal in breast reconstruction.

A number of studies have reported, through small and medium series of patients, on the safety of tissue replacement techniques during oncoplastic lumpectomy.^3,5,6,8,15,17–20,23–26^ With regards to the oncological safety of these flaps, authors have reported different management of positive margins post oncoplastic reconstruction using tissue replacement techniques. Meybodi et al had one positive margin managed with reexcision.^23^ For Agrawal et al., five patients had positive margins, with one cavity shave and one completion mastectomy.^27^ In our study, we had one positive margin that occurred in a patient treated with mastectomy. After thorough multidisciplinary review, it was thought that maximal resection was already achieved and the decision was made to complete her treatment with radiation alone. Our goal for this study was to highlight the safety of these flaps and to encourage surgeons to consider its role in extending the options for breast conservation as well as salvage from failed reconstruction or specific cases of mastectomy reconstruction.

In general, lateral chest wall flaps have a number of distinct advantages including preservation of the underlying latissimus muscle which can still be used if needed in the future, a relatively well concealed scar, the avoidance of a contralateral balancing procedure, the ability to perform the resection and reconstruction in the same position.^27^ In the case of mastectomy reconstruction, free flaps and standard alloplastic techniques are effective but severely resource or material intensive. The ability to use a local flap when appropriate can alleviate some of these resource pressures.

Larger resections are generally possible with LICAP flaps as compared with other chest wall perforator flaps.^8^ Our average tumor size was 33.8 mm and lumpectomy excision volume was 113 g. With regard to tumor location, the majority of our patients had lateral or central involvement (13 patients). This is naturally an easier location to fill with these flaps that are laterally based and is likely an option in any patient. Seven patients had medial breast involvement that could also be filled with a lateral chest wall flap. In this more difficult area, considerations including overall breast size, tumor size, and volume of flap available will determine whether a lateral chest wall flap is appropriate. From our first experience with this flap, it was easy to learn, versatile, and robust. Over time, a number of important considerations have been incorporated. We have seen, for example, accurate and efficient intraoperative perforator identification by placing the patient in the simulated surgical position for marking and for doppler vessel identification preoperatively as described by Hamdi.^17^ The modification in flap marking along with the modified scar position (Figure 7) has allowed for larger volume flaps and more hidden scar. Placement of the patient on an inflatable bolster or beanbag allows for a reasonable position for the excisional surgery, sentinel node surgery, and flap dissection while deflation of the bolster intraoperatively allows the patient back into a supine position, facilitating more accurate flap inset.

Previous reports have described the outcomes from intercostal artery flaps. In some cases, these are performed by oncoplastic surgeons that perform both the resection and reconstruction.^15–17^ One such study reports a positive margin rate of 13.4% with this approach.^16^ At our center, we feel strongly that these cases should be managed as a multidisciplinary team with the oncologic breast surgeon working together with the plastic surgeon; each focused on their primary goal—oncologic clearance versus reconstruction of an aesthetically pleasing breast mound. With this team approach, we were able to avoid a positive margin in all lumpectomy patients. These procedures were all done as day surgery procedures (also as compared to Soumian that kept patients for a median of 1 day).

An exceedingly low overall donor site complication rate of 3.7% has been reported for all chest wall perforator flaps.^8^ We had no recorded donor site complications. Flap overall complications in a recent systematic review of 13 articles was 9.25% and 11.84% harvesting complications.^8^ The sample size of the studies included was 8 to 87 patients, with 1 to 8 complications in each study. In this current study, the utility of the chest wall flap was significantly extended by combining the flap with alloplastic reconstruction or volume rearrangement techniques. Even with this greater heterogeneity, our flap complication rate was only 7.7%. These low complication rates may also be related to the 2-team approach with the Plastic Surgeon focused on the flap reconstruction.

### Limitations

The primary purpose of this report is to highlight the versatility of the lateral chest wall flaps in breast reconstruction. This study includes a small sample number which precludes the ability to make conclusions that apply to broader populations. The focus of this study was meant to be with regard to versatility and safety within these specific patients. Another limitation of this study is its retrospective nature. Only available data were included in this manuscript. Though missing data was minimal, it remains a limitation of retrospective studies. The relatively short follow-up period of 8.5 months is long enough to claim an exceedingly low short-term complication rate. We are not, however, able to make conclusions regarding long-term oncologic or aesthetic outcomes. Breast conserving surgery with or without previously described oncoplastic techniques has been shown to have excellent oncologic outcomes. Given that the excisional component of the procedures we describe is not different, we would not expect any difference in long-term oncological outcomes. This short follow-up may also miss patients who are still contemplating. Although we did not formally collect patient reported outcomes, we note, anecdotally, that patients that underwent chest wall flap reconstruction were very satisfied in their postop follow-ups. This has been noted in recent studies examining cosmetic outcomes with chest wall flaps.^8^ A further study looking specifically at patient reported outcomes with the use of lateral chest wall flaps is clearly necessary.

## Conclusions

The use of lateral chest wall flaps can significantly extend the options for breast reconstruction with minimal donor site morbidity. In many cases, these flaps avoid what would have otherwise been a mastectomy and reconstruction involving more resource and morbidity. In cases, where a mastectomy is still necessary, these flaps can be used to provide for a reasonable breast mound in patients that are otherwise not good candidates for other types of reconstruction. These flaps are uniquely advantageous in that they preserve the Latissimus Dorsi flap for salvage when needed. Our approach included a breast surgeon and plastic surgeon working together in all cases. This, we believe, is responsible for our low positive margin rate, low complication rate, and low revision rate. Our experience demonstrates ease of use and versatility and provides support that lateral chest wall flaps could be a valuable piece of any surgeon's reconstructive arsenal.
